# MAF1 Sequesters Mitochondria for *Toxoplasma gondii*

**DOI:** 10.1371/journal.pbio.1001846

**Published:** 2014-04-29

**Authors:** Caitlin Sedwick

**Affiliations:** Freelance Science Writer, San Diego, California, United States of America

Mitochondria, the powerhouses of eukaryotic cells, regulate many pathways related to cell growth and division. They also participate in apoptotic cell death and survival pathways such as autophagy and in innate immune responses. As such, mitochondria are a tempting target for intracellular pathogens, which may seek to block or manipulate mitochondrial activity to meet their own needs.

**Figure:**
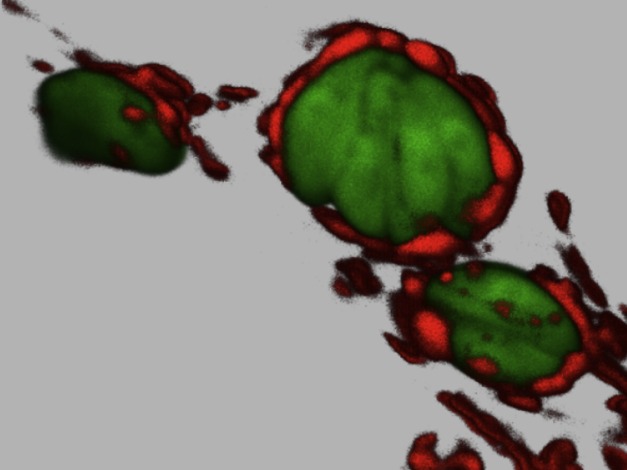
Human fibroblasts infected with Type I *Toxoplasma gondii* (green) recruit host mitochondria (red) to the vacuole in which they grow. *Image Credit: Lena Pernas, Stanford University.*

Sometimes it is obvious when a pathogen is affecting mitochondria. For example, the protozoan *Toxoplasma gondii* is a parasite that resides within intracellular vacuoles inside host cells. Some strains of *T. gondii* can corral and sequester their host's mitochondria together at the vacuole—a process termed host mitochondrial association (HMA). How the parasite achieves HMA, and what benefits it gains from doing so, are unknown. But now, as they report in their paper published this month in *PLOS Biology*, Lena Pernas, John Boothroyd, and colleagues have brought us closer to understanding the mechanism used by *T. gondii* to sequester host cell mitochondria.

Pernas and colleagues began by examining three *T. gondii* strains known to cause disease in humans, which are classified by type (type I, II, and III) according to their genetic lineage. The three strains are different genetically and in many other respects; for example, each strain provokes a qualitatively different response from infected individuals' immune systems. This characteristic led the authors to wonder whether the different strains might also behave differently with respect to HMA. To test this, Pernas and colleagues exposed human foreskin fibroblasts to the separate strains and then looked for evidence of mitochondrial sequestration. Importantly, both the type I and type III strains caused HMA, but the type II strain, Me49, did not. The absence of HMA appears to be a feature typical of type II *T. gondii* parasites, because another type II strain also failed to induce HMA.

To determine the mechanistic basis for HMA, Pernas and coworkers evaluated the progeny of a cross between type II and type III parasites for their ability to induce HMA. Then, the strains' HMA status was compared with their possession of different chromosomal markers, to determine which chromosomal region hosted the gene necessary for HMA. The region identified contained 153 candidate genes, and the authors then narrowed the field further by searching this portion of the parasite's genome for genes with features likely to be important for HMA. For example, Pernas and colleagues suspected the gene they sought would probably encode a membrane-spanning protein whose expression levels differed between HMA-positive and HMA-negative strains. Using this approach, the researchers ultimately identified one gene, designated as *TGG1_053770* in the *T. gondii* genome database, as a likely candidate to mediate HMA.

Pernas and coworkers next examined the protein's location in *T. gondii*-infected host cells using immunofluorescence. The protein was observed lining the limiting membrane of the vacuole that houses type I and type III parasites, but not that of type II parasites. Western blotting showed that both type I and type III parasites expressed large amounts of *TGG1_053770*'s protein product, but type II parasites did not. These data raised the possibility that *TGG1_053770* may encode a protein important for HMA, so the authors tentatively dubbed it mitochondrial association factor 1 (MAF1).

To determine if MAF1 really does mediate HMA, Pernas and coworkers genetically engineered type II parasites to express the version of MAF1 found in the type I strain. Indeed, the modified type II parasites now recruited host mitochondria, whereas type I parasites whose MAF1 gene was deleted could not. Interestingly, MAF1-deficient type I parasites grew at about the same rate as their wild-type counterparts and, similarly growth of type II parasites was unaffected by the presence or absence of MAF1.

Although Type II parasites don't require MAF1 to grow, their expression of MAF1 causes dramatic changes to host cell biology. First, Pernas and colleagues observed that host mitochondria infected by wild-type type II parasites are not sequestered and appear normal in size. In contrast, those sequestered by MAF1-expressing type II parasites expanded to a huge size. MAF1 also altered host cells' response to parasite infection; murine monocytes growing in cell culture secreted a different constellation of immunomodulatory proteins when infected with MAF1-expressing type II versus wild-type type II parasites. Finally, in mice infected with parasites, the presence or absence of MAF1 affected both the host animals' immune responses and the patterns of genes they expressed in response to infection.

Together, these results suggest that MAF1 is involved in *T. gondii* HMA, and that MAF1 affects the course of *T. gondii* infections. At this time, the mechanisms by which MAF1 drives HMA remain unclear, and the impact MAF1 has on mitochondrial biology is also unknown. The identification of MAF1 as the mediator of *T. gondii* HMA will help scientists answer these questions and many more.

**Pernas L, Adomako-Ankomah Y, Shastri AJ, Ewald SE, Treeck M, et al. (2014)** ***Toxoplasma*** **Effector MAF1 Mediates Recruitment of Host Mitochondria and Impacts the Host Response.** doi:10.1371/journal.pbio.1001845

